# Impact of calcium nitrate supplementation on the oxygen-carrying capacity of lactating sows and their offspring

**DOI:** 10.1093/tas/txab217

**Published:** 2021-11-14

**Authors:** Jennifer L G van de Ligt, Kari L Saddoris-Clemons, Sharon A Norton, Meagan M Davis, Candace L Doepker

**Affiliations:** 1 ToxStrategies, Inc., Brooklyn Park, MN 55443, USA; 2 Department of Veterinary Population Medicine, College of Veterinary Medicine, University of Minnesota, St. Paul, MN 55108, USA; 3 Wayzata, MN 55391, USA; 4 ToxStrategies, Inc., Katy, TX 77494, USA; 5 ToxStrategies, Inc., Newport, KY 41071, USA

**Keywords:** lactation, methemoglobinemia, nitrate, piglets, sows

## Abstract

Calcium nitrate supplementation has recently been suggested to provide potential benefits to sows and, in particular, their offspring when administered at a level of 1,200 ppm in feed shortly before farrowing through lactation. More specifically, nitrate supplementation has been suggested as one opportunity for improved placental and/or fetal blood flow and has been hypothesized in previous work to be important to the swine industry in light of the global trend toward larger litter sizes. The benefit is likely manifested through exposure to the nitrate moiety, but interestingly, nitrate has historically been considered a compound of concern for swine. High levels of nitrate once metabolized to nitrite can interfere with the oxygen-carrying capacity of hemoglobin, resulting in increased methemoglobin and, subsequently, methemoglobinemia (MetHb) if the animal is deprived of significant amounts of oxygen; however, the level of nitrate exposure necessary to induce MetHb in sows is not clearly defined. This work was undertaken to examine methemoglobin levels in sows and piglets exposed to the potentially beneficial levels of 1,200 and 6,000 ppm nitrate added to their diets over the course of the periparturient period. Other oxygen capacity blood variables were evaluated (e.g., hemoglobin, hematocrit, and various measures of hemoglobin and red blood cell volumes and concentrations), as well as performance endpoints (weight changes and feed intake) and general observations over the 27-d period. No evidence of treatment-related toxicity manifestation was observed at these supplemental levels. Nearly all oxygen-related variables were affected by time (independent of treatment), indicating adaptive general effects of farrowing. These findings support the hypothesis that MetHb is not a concern up to at least 6,000 ppm supplemental nitrate exposure, even in combination with additional nitrate in the sow’s daily diet. This work is important to help swine producers understand that consideration of nitrate benefit should outweigh concern for risk of nitrate-induced toxicity.

## INTRODUCTION

Numerous reviews of the potential beneficial physiological effects related to dietary nitrate intake in humans have been conducted, and Bryan and Loscalzo (2017) have published a book that summarizes these findings. Beneficial effects from dietary nitrate relate mainly to improved vascular function, which occurs because dietary nitrate is a nitric oxide (NO) precursor (Lundberg and Govoni, 2004), and NO is an endothelium-derived relaxing factor that produces vasodilation. In swine, this type of vascular-related physiological effect can play a beneficial role in regulating placental–fetal blood flow and transfer of nutrients and oxygen from mother to fetus (Bird et al., 2003). The potential improvement of placental fetal blood flow has been hypothesized to be important to the swine industry in light of the global trend toward larger litter sizes (Prunier et al., 2010; Knauer and Hostetler, 2013; van den Bosch et al., 2019a, b). In the last days of pregnancy, fetal growth increases dramatically and placental capacity and efficiency become even more important as feto-placental units may be compromised due to uterine crowding (Kemp et al., 2018). These larger litter sizes frequently result in the potential for increased numbers of stillborn piglets and lower piglet vitality, due to a variety of factors including, but not limited to, a reduction in exchange of blood flow and oxygen between the mother and the fetuses during the long process of farrowing (Prunier et al., 2010; Kemp et al., 2018; Udomchanya et al., 2019; van den Bosch, 2019a, b). In addition, larger litter sizes have been shown to result in higher levels of circulating cortisol during late pregnancy and parturition (Wähner et al., 2005; Roelofs et al., 2019). This higher level of circulating cortisol in sows with larger litters may further impinge uterine blood flow due to cortisol increasing vascular sensitivity and vasoconstriction from epinephrine and norepinephrine, which also increase dramatically immediately before and during parturition (Eliot et al., 1981; Roelofs et al., 2019). Thus, when considering preliminary evidence in rats and humans where cortisol suppressed the nitric oxide system important for vasodilation and dietary nitric oxide precursors alleviated the suppression, it is possible that sows, especially those with larger litter sizes, may benefit from dietary nutrient precursors of nitric oxide in order to maintain adequate uterine blood flow in late gestation and during parturition to assure normal oxygenation and vitality of piglets (Herpin et al., 1996; Kelly et al., 1998). In fact, sow diets that contain 900 to 1,200 ppm supplemental nitrate have been shown to support a sufficient, oxygen-rich blood supply to the uterus and reduce instances of stillborn piglets (van den Bosch, 2019a, b).

Previous reviews have given rise to the notion that dietary nitrate in feed or water has traditionally been considered an unwanted contaminant, with a potential concern for nitrate toxicity in swine (Smith et al., 1959; Emerick, 1974; Bruning-Fann and Kaneene, 1993; Nyachoti and Kiarie, 2010). Nitrate can be converted to nitrite by microflora. Absorbed nitrite can then combine with red blood cells where it oxidizes the ferrous iron in hemoglobin, forming methemoglobin. Methemoglobin has compromised oxygen carrying capacity (heme is unable to bind to oxygen) and reduces blood oxygenation and, eventually, can induce tissue hypoxia. Work by Bhattarai et al. (2019) indicated that hemoglobin measurements in both sow and piglet can be used as indicators for performance of the piglets later in life. In humans, a level of 10% methemoglobin, as a percentage of total hemoglobin, has been shown to be the threshold for toxicity in the blood, and this threshold is expected to be similar in swine (Wright et al., 1999; Thompson, 2014).

Although swine production feeding practices vary, it can be said that swine are exposed to nitrate through various feedstuffs. Conclusions from a recent comprehensive weight-of-evidence evaluation on the existing literature corroborated EFSA’s safety benchmark value for nitrate of 410 mg/kg-bw/d (EFSA, 2020) in swine as protective of concerns for MetHb as well as for adversity related to vitamin A depletion, growth, and reproductive performance. Moreover, the application of the weight-of-evidence evaluation provided moderate-to-high confidence that the no-observed-adverse-effect level (NOAEL) is likely above EFSA’s value and between 674 and 870 mg/kg-bw/d (~600 to 800 mg/kg-bw/d; Doepker et al., 2021).

The objective of this study was to investigate the relationship between supplemental nitrate and the likelihood of MetHb occurrence. We hypothesized that dietary supplementation of up to 6,000 ppm nitrate during the periparturient period would be well tolerated by sows and not be likely to induce any signs of MetHb or any other negative impacts on sows or their offspring.

## MATERIALS AND METHODS

To confirm the hypothesized NOAEL of supplemental dietary nitrate in sows, a targeted dosage of 1,200 ppm calcium nitrate and a 5× dosage (6,000 ppm) were chosen to ensure a substantial margin of safety were fed from placement into the farrowing rooms and continued throughout lactation. Calcium nitrate dihydrate (calcium nitrate; CAS No. 10124-37-5) was utilized as the source of dietary nitrate. The target dosage of 1,200 ppm nitrate was chosen from the optimal dosage reported by van den Bosch et al. (2019a, b). This study was conducted at the Cargill Velddriel Research Facility in the Netherlands. Animal care approval was granted consistent with the scope of the Institutional Animal Care and Use Committee (IACUC) AVD2200020171646 for pig biomarkers and met EU Feed Regulation EC No. 1831/2003. All work was conducted under typical commercial production conditions during the study period, using measurements of methemoglobin in the blood and clinical signs of the development of MetHb in sows and piglets as the metric of adverse effect.

### Animals

A total of 24 sows (Yorkshire × Dutch Landrace; Topigs 20), ranging in parity from 1 to 7 and mated with Topigs Pietrain boars were included in the study. Females were placed into individual farrowing crates on day −1, approximately 4 d prior to farrowing, and remained there until weaning of piglets resulting in a lactation period of 21 to 25 d. Pens were 4.4 m^2^, with a combination slatted and solid floor. Each pen contained a single-space feeder for the sow, a feeding bowl for the pigs, a single drinking nipple, a hanging heater, and enrichment toys for both sows and pigs.

Animals were managed according to standard procedures for routine care, with the exception that no creep feeds were supplied to the piglets. All animals were observed twice daily for any health abnormalities, including specific attention to activity and breathing rate as an indicator of potential nitrate toxicity. In the event of altered activity or breathing rate, procedures were in place for further evaluation of mucous membranes and consultation with the attending veterinarian.

### Study Design

This study was a completely randomized block design. The farrowing unit consisted of two rooms (Room A and Room B), with 12 crates per room. Room A housed six parity 1 gilts in block 1 and three parity 5, one parity 6, and two parity 7 sows in Block 4. Room B housed four parity 3 and two parity 4 sows in block 2 and six parity 2 sows in block 3. Each treatment was present twice in each block.

On d −1, sows were moved into the farrowing unit, weighed, and had backfat measurements collected. Sows were blocked by parity and randomly assigned to one of three dietary treatments within block: 0 ppm supplemental nitrate (control diet), 1,200 ppm supplemental nitrate, and 6,000 ppm supplemental nitrate. Sows received a basal lacatation diet containing no calcium nitrate on day −1. On the morning of day 0, blood samples were taken and the sows were then placed on dietary treatments. The sows were limit fed at 3.2 kg/d prefarrowing, and gilts were fed 2.8 kg/d. Postfarrowing feeding amounts were increased and individualized. Lactation feed amounts started at 2 kg/d postfarrowing and were increased by 0.5 kg/d to a maximum of 8 kg/d. The sows were fed twice per day, and feed intake and feed refusal were monitored daily to adjust the feeding schedule.

The sows were evaluated during two periods: 1) from entering the farrowing pens until all sows were farrowed, and 2) from cross-fostering to weaning. In the event that a litter contained more than 14 pigs, split suckling was implemented during the first 24 h postfarrowing. At 24 h postfarrowing, cross-fostering of pigs was implemented within treatment groups following placement of individual ear tags and measurement of the total number of liveborn, stillborn, and individual pig weights. On day 7, pigs were weighed individually to evaluate the weight distribution across blocks within treatment, and to correct if necessary. Pig performance was evaluated from cross-fostering to weaning.

### Treatment Diets

The same dietary ingredients were used for each formulation, and calcium in each formulation was corrected for the addition of calcium nitrate (CAS No.: 10124-37-5 (Ca(NO_3_)_2_ 2.2H_2_O); Adob, batch B: 1024/20/08, production date May 13, 2020). Diets manufactured by ABZ at facilities in Nijkerk and Leusden, both in the Netherlands, were provided as 4 mm diameter pellets. All diets were formulated to meet or exceed National Research Council Nutrient Requirements of Swine for gestating and lactating sows (NRC, 2012). The dietary formulation and calculated and actual nutrient content of the treatment diets are presented in Table 1.

**Table 1.** Dietary treatment ingredient composition (%)

Ingredient	Treatment A—0 ppm nitrate	Treatment B—1,200 ppm nitrate	Treatment C—6,000 ppm nitrate
Diet formulation			
Cereal grains	47.50	47.41	47.04
Vegetable proteins	25.70	25.70	25.70
Roughages	17.90	17.90	17.90
Molasses	2.50	2.50	2.50
Fats and oils	2.89	2.89	2.89
Phytase	0.003	0.003	0.003
VTM premix	0.90	0.90	0.90
Limestone	1.37	1.28	0.89
Monocalcium phosphate	0.51	0.51	0.51
Salt	0.40	0.40	0.40
Sodium bicarbonate	0.20	0.20	0.20
l-Lysine	0.12	0.12	0.12
Calcium nitrate	0.00	0.19	0.95
Nutrient composition (calculated)			
NE, kcal/kg	2,399	2,397	2,387
Nitrate, %	0	0.12	0.60
Crude fat, %	5.22	5.22	5.22
Crude protein, %	16.8	16.9	17.6
SID lysine, %	0.76	0.76	0.76
Calcium, %	1.10	1.10	1.10
Available phosphorus, %	0.33	0.33	0.33
Actual nutrient ^1^composition			
Nitrate, %	0.0022	0.1259	0.6297
Nitrite, %	<0.0005	<0.0005	<0.0005
Crude protein, %	17.23	17.07	16.80
Fat, %	4.60	4.61	4.66
Fiber, %	6.98	7.17	7.10
Moisture, %	11.92	12.11	12.67
Dry matter, %	88.10	87.90	87.30
Ash, %	5.00	5.12	4.92
Calcium, %	0.963	1.100	1.100

Crude protein, fat, fiber, moisture, and ash provided for analysis of feed were performed using Near Infrared spectroscopy. Calcium was analyzed using X-ray fluorescence spectroscopy. Feed, raw material and foodstuff nitrate concentrations were analyzed according to ANAL-10147. All analytical testing was performed by NutriControl B.V. in the Netherlands.

### Blood Collection and Analysis

Blood samples were collected from all sows on day 0 in the morning (following entry into farrowing crates day 0, as well as on days 12 and 25. Samples were collected via jugular venipuncture into various tubes appropriate for the blood analytical analysis: heparin-treated tubes for blood gas and methemoglobin values and EDTA-treated tubes for hematology.

Blood samples were collected from the four largest piglets in each litter on day 13, which corresponded to an average of 7 d of age for the piglets (range 4 to 8 d of age). On day 27 (the day of weaning), blood was collected from the two largest piglets due to equipment failure of the Radiometer ABL90 Flex Plus blood gas analyzer on day 26. The equipment failure reduced the number of available heparin tubes were available to obtain blood samples on day 27 when functional analytical equipment was procured. This resulted in a deviation from the sampling plan, reducing the number of blood samples collected, due to low methemoglobin stability, from four to two piglets per litter. Blood samples for methemoglobin measurement, were collected via jugular venipuncture into heparinized tubes.

Blood collected for blood gas and methemoglobin measurement was analyzed immediately with a Radiometer ABL90 Flex Plus blood gas analyzer, except on day 27 when a Rapidpoint 500 blood-gas analyzer was used. Blood for hematology measurements was cooled and sent for same-day measurement at Euregio Laboratory Service, Maastricht.

Samples for hematology (hematocrit (vol%), hemoglobin (mmol/L), and red blood cell quality (mean corpuscular volume [MCV] of erythrocytes, mean corpuscular hemoglobin [MCH], and mean corpuscular hemoglobin concentration [MCHC]) were analyzed by flow cytometry.

### Statistical Analysis

All data were analyzed in R (version 3.6.1), deploying packages lme4 (version 1-21) for general linear model implementation and the “poly” function of the stats package (version 3.6.0) for contrast coefficient generation. The confint() method was used to generate a 95% confidence interval for the linear slope of response of MetHb to dietary nitrate level.

Sow performance metrics were analyzed as a completely randomized block design with supplemental nitrate levels included as treatment. Sow was the experimental unit and confounded effect of sow parity and location was included as a random blocking factor. The statistical model for sow performance was: *Y*_*ijk*_ = *µ* + *τ*_*i*_ + *b*_*j*_ + *e*_*ijk*_, where *µ* = overall mean, *τ*_*i*_ = fixed effect of dietary treatment, *b*_*j*_ = random effect of confounded parity and location block, and *e*_*ijk*_ = random error associated with the *e*_*ijk*_th observation. Sow body weight and backfat at entry to the farrowing barn and exit at weaning, sow average daily feed intake (ADFI), average birth weight of liveborn piglets, and average total birthweight, were analyzed using a general linear model. Count data for the number of live born, stillborn, and weaned piglets, as well as mortality after cross-fostering were analyzed using a probabilistic generalized mixed model specifying a binomial distribution with a logit link. Inference on LSMeans was made using linear and quadratic contrasts to further examine the effect of dietary calcium nitrate level on sow performance.

The sow and piglet blood methemoglobin data were analyzed as a completely randomized block design with a factorial arrangement of treatments, including supplemental nitrate level and time as factors. The statistical model for sow methemoglobin data is as follows: *Y*_*ijkl*_ = *µ* + *τ*_*i*_ + *α*_*j*_ + (*τ* × *α*)_*ij*_ + *b*_*k*_ + *e*_*ijkl*_, where *µ* = overall mean, *τ*_*i*_ = fixed effect of dietary treatment, *α*_*j*_ = fixed effect of time of assessment, (*τ* × *α*)_*ij*_ = fixed interaction effect of dietary treatment by time of assessment, *b*_*k*_ = random effect of confounded parity and location block, and *e*_*ijkl*_ = random error associated with the *e*_*ijkl*_th observation. Piglet blood samples were included as sub-samples from each sow, where sow was the experimental unit. The statistical model used was: *Y*_*ijklm*_ = *µ* + *τ*_*i*_ + *α*_*j*_ + (*τ* × *α*)_*ij*_*b*_*k*_ + *c*_*l*_ + *e*_*ijkm*_, where *µ* = overall mean, *τ*_*i*_ = fixed effect of dietary treatment, *α*_*j*_ = fixed effect of time of assessment, (*τ* × *α*)_*ij*_ = fixed interaction effect of dietary treatment by time of assessment, *b*_*k*_ = random effect of confounded parity and location block, *c*_*l*_ = random effect of the *l*th piglet blood subsample on the *e*_*ijkm*_th sow, and *e*_*ijkm*_ = random error associated with the *e*_*ijkm*_th observation. Sow and piglet MetHb blood levels were analyzed as a general linear model with fixed and random effects. Scaled unequally spaced orthogonal polynomial contrasts were established to test the hypothesis of a linear slope of response to dose slope being equal to zero (no slope), and their estimates were used to establish the linear slope relationship (value of slope of response to dose). To accommodate the continuous nature of the effect of time, inference on LSMeans was made using linear and quadratic contrasts to further examine the effect of dietary calcium nitrate level, time, and their interaction on blood MetHb.

## RESULTS AND DISCUSSION

No physical signs of adverse effects (e.g., changes in activity/breathing rates) were noted in any sows or piglets during the study period. There were no significant changes in dietary intake, and no clinical signs of nitrate poisoning were observed during the study. All sows tolerated dietary nitrate supplementation at levels up to 6,000 ppm.

### Feed Consumption and Supplemental Nitrate Intake

Dietary treatment of supplemental nitrate had no effect (*P* > 0.10) on feed consumption by the sows in the pre- or post-farrowing periods (Table 2). The sows fed 1,200 ppm supplemental nitrate received the pre-farrowing diet approximately 1 d less than the other treatments due to timing of allotment and farrowing, and the post-farrowing diet for an additional 1.2 d, as compared to the control diet (0 ppm) and 6,000 ppm treatments.

**Table 2.** Effect of dietary nitrate supplementation on pre- and post-farrowing average daily feed intake (ADFI)

Variable	Treatment means			SEM	Contrast prob *F*	
	0 ppm	1,200 ppm	6,000 ppm		Linear	Quadratic
Pre-farrowing ADFI^1^, kg/d	2.965	2.900	2.817	0.194	0.608	0.891
Post-farrowing ADFI2, kg/d	5.919	5.758	5.749	0.373	0.855	0.702

^1^Sows averaged 4.25, 3.13, and 4.38 d on feed pre-farrowing in the 0, 1,200, and 6,000 ppm treatment groups, respectively.

^2^Sows averaged 22.75, 23.88, and 22.6 d on feed post-farrowing in the 0, 1,200, and 6,000 ppm treatment groups, respectively.

The combination of feed consumption, days in each period, and nitrate intake for the targeted treatment levels of the control diet (0 ppm; actual 22 ppm), 1,200 ppm (actual 1,260 ppm), and 6,000 ppm (actual 6,300 ppm) resulted in average daily nitrate intakes of 65.2 mg, 3,654 mg, and 17,747 mg, respectively, in the pre-farrowing period (time period approximately 3 d). Correspondingly, the post-farrowing period of approximately 24 d resulted in an average daily nitrate intake of 130 mg, 7,255 mg, and 36,219 mg, respectively. Expected and actual treatment diet composition can be found in Table 1. The study design was intended for the 6,000 ppm treated sows to consume 5 times the amount of nitrate as the 1,200 ppm sows. The intended supplemental consumption difference was almost achieved in the pre-farrowing period (4.9 times) and was achieved in the post-farrowing period (5 times) (Table 2).

### Sow and Piglet Methemoglobin Measurements

After oral exposure, nitrate is reduced to nitrite, typically by oral bacteria; nitrite is subsequently reduced to nitric oxide. Nitrite ions in the blood that come in contact with red blood cells oxidize ferrous iron found in hemoglobin to the ferric state, creating methemoglobin, which cannot appropriately carry oxygen. Methemoglobin concentration is a reliable indicator of excess nitrate or nitrite exposure only under conditions of acute toxicity (Thompson, 2014). In humans, Wright et al. (1999) suggested that the acute toxicity resulting from the presence of methemoglobin without the manifestation of MetHb has a threshold of greater than 10% methemoglobin in the blood, and this threshold is expected to be similar in swine.

Sows consuming higher levels of nitrate tended to have higher levels of methemoglobin in their blood in a dose-dependent manner (treatment, linear *P* < 0.10) and when fed for longer periods of time (time, linear *P* < 0.005; Table 3). Sows consuming 6,000 ppm supplemental nitrate had an average methemoglobin level of 1.30%, the highest recorded level of 3.6% methemoglobin in an individual sow, and all were well below the 10% toxicity threshold value.

**Table 3.** Effect of dietary nitrate supplementation on blood measurements in sows

Variable	Treatment means			SEM	Contrast prob *F*					
	0 ppm	1,200 ppm	6,000 ppm		Treatment		Time		Treatment × time	
					Linear	Quadratic	Linear	Quadratic	Linear	Quadratic
Methemoglobin, vol%										
Day 0	0.925	0.813	0.713	0.166	0.068	0.315	0.002	0.444	0.119	0.916
Day 12	0.650	0.887	1.252							
Day 25	0.975	1.300	1.304							
Hematocrit, vol%										
Day 0	40.0	37.8	37.3	1.50	0.263	0.202	<0.0001	<0.0001	0.201	0.944
Day 12	39.8	38.7	38.6							
Day 25	27.6	27.5	28.0							
Hemoglobin, mmol/L										
Day 0	7.26	6.74	6.60	0.283	0.157	0.150	0.100	0.065	0.202	0.882
Day 12	7.17	6.88	6.91							
Day 25	6.59	6.57	6.60							
Mean corpuscular value (MCV), fL										
Day 0	73.2	72.59	73.51	1.55	0.981	0.235	<0.0001	<0.0001	0.694	0.969
Day 12	74.95	73.98	74.46							
Day 25	53.15	51.81	52.41							
Mean corpuscular hemoglobin (MCH), fmol										
Day 0	1.34	1.29	1.31	0.034	0.572	0.009	<0.0001	<0.0001	0.465	0.678
Day 12	1.35	1.32	1.33							
Day 25	1.26	1.24	1.26							
Mean corpuscular hemoglobin concentration (MCHC), mmol/L										
Day 0	18.29	17.78	17.77	0.291	0.412	0.251	<0.0001	<0.0001	0.145	0.781
Day 12	18.03	17.89	17.89							
Day 25	23.75	23.85	23.91							

**Figure 1.**
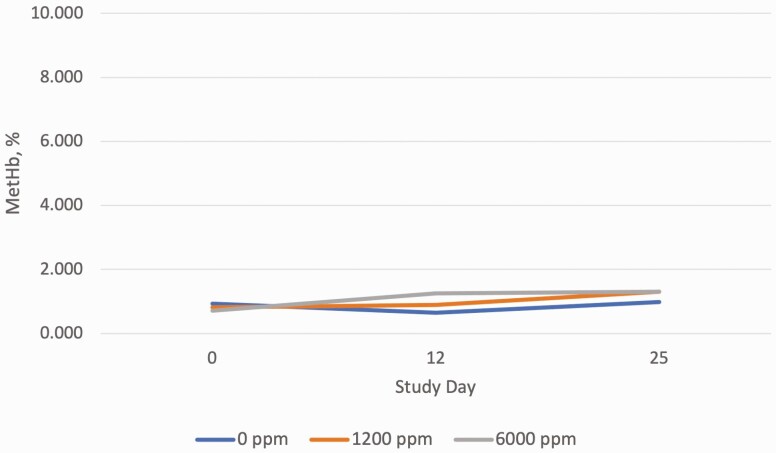
Methemoglobin response to nitrate supplemental concentration in feed during days 0 to 25 of lactation. SEM = 0.166 Treatment effect: linear, *P* = 0.068; time effect: linear, *P* = 0.002.

To further explore the relationship between time and nitrate supplementation on methemoglobin levels, a linear slope analysis was conducted, using an orthogonal polynomial contrast (Figure 1). The slope of 0.1518 tended to be different from zero (*P* < 0.10) indicating that there is a tendency for methemoglobin levels to slowly linearly increase over time as the level of nitrate in the diet increases. The 95% confidence interval for this linear slope was −0.0118 to 0.3254 indicating the slope uncertainty at *α* = 0.05, the range includes a slope of zero (no slope).

No effect of maternal dietary nitrate supplementation treatment was noted for piglet methemoglobin blood levels (*P* > 0.10; Table 4). Piglets across all treatments had higher methemoglobin levels at weaning than at 14 d of age (*P* < 0.0001). No time × treatment effects were observed (*P* > 0.10). The highest recorded methemoglobin level for any piglet was less than 1.6%, well below the threshold of concern of 10% discussed above.

**Table 4.** Effect of dietary nitrate supplementation on serum methemoglobin, %, in piglets

Variable	Treatment means			SEM	Contrast prob *F*				
	0 ppm	1,200 ppm	6,000 ppm		Treatment		Time	Treatment × time	
					Linear	Quadratic	Effect	Linear	Quadratic
Day 13	0.507	0.504	0.520	0.082	0.235	0.185	<0.0001	0.325	0.208
Day 27	0.817	0.669	0.900						

### Sow Blood Measurements

Reference values of typical hematological measurements (Table 5) were included for comparison and discussion. A few authors have noted that it is often difficult to find reference values for gestating sows (Perri et al., 2017; Bhattarai et al., 2019), despite such reference values being valuable for veterinarians and researchers. There can be variance across geographic regions and between herds, hence these values should be used to provide perspective on physiological changes versus clinical or toxicity events (none of which occurred). Present findings indicate that most of the measured hematological variables were within expected physiological ranges.

**Table 5.** Various reference values of blood variables in swine in comparison to reported range of data

Value of interest (units)	Abbreviated description	Merck index	Perri et al. (2017) range	Bhattarai et al. (2019) range	Study min and max of means^1^
Hemoglobin (mmol/L)	Oxygen carrier in blood	6.21–9.93	5.65–8.94	6.3–10.5	6.57–7.26
Serum hematocrit (%)	Volume percentage of RBC in blood	36.0–43.0	30.0–50.0	32.0–55.0	27.50–40.00
Mean corpuscular volume (fL)	Avg size and volume of RBC	50.0–68.0 fL	48.0–87.0	55.6–70.2	51.81–74.95
Mean corpuscular hemoglobin (fmol)	Measure of hemoglobin content in individual RBC	1.06–1.30	0.81–1.68	1.08–1.36	1.24–1.35
Mean corpuscular hemoglobin concentration (mmol/L)	Measure of hemoglobin concentration in individual RBC	18.62–21.10	16.26–24.64	14.85–21.02	17.77–23.91

^1^Range of means for all treatment groups; values not reported by treatment group because there was no treatment effect.

Hemoglobin is responsible for carrying oxygen in the blood, which was hypothesized to decrease with nitrate supplementation, based on the mechanism of nitrite inhibiting oxygen-carrying capacity. Sow data indicated a significant quadratic effect on hemoglobin levels with time (*P* < 0.10; Table 3), not treatment. Interestingly, the largest change between average hemoglobin values at the three different time points was within the control group. Quadratic changes in hemoglobin levels over the periparturient period would be expected with normal blood loss occurring at farrowing followed by increasing hemoglobin levels with time following parturition (Castevens et al., 2020). This impact of farrowing as the primary driver for blood hemoglobin levels is further supported by the control group values having the largest change in average values over time.

Hematocrit, a measure of the red blood cell content, was significantly different over time only (not treatment) with both linear and quadratic effects (*P* < 0.0001). This decrease was consistent across all three groups, suggesting that this was a normal periparturient change. Sows in this study had serum hematocrit levels at the lessor values of the expected range at the end of the study and no evidence of impact on performance was noted.

The mean cell or corpuscular volume (MCV), a measure of red blood cell size, was consistent with the other findings, in that the data indicate significant changes over time but not with treatment. The mean corpuscular hemoglobin (MCH) value indicates how much hemoglobin is in each red blood cell.

Sows fed 1,200 ppm supplemental nitrate had lower MCH levels than sows receiving either 0 or 6,000 ppm supplemental nitrate, indicating a significant treatment quadratic effect (*P* < 0.01). Additionally, a significant time effect on MCH was noted with both linear and quadratic effects (*P* < 0.0001). While MCH was significantly lower for sows exposed to 1,200 ppm nitrate, levels were still well within the defined normal range.

The mean corpuscular hemoglobin concentration (MCHC) reflects the average amount of hemoglobin per unit volume. These levels were not affected by dietary nitrate supplementation (*P* > 0.10) but were significantly affected by time with both linear and quadratic effects (*P* < 0.0001). Because MCHC is a reflection of hemoglobin, this finding is not surprising and is consistent with the hemoglobin data discussed above which was also affected with by time. Sow MCHC levels were at the lower end of the expected range at the start of the study. Despite this observation, no evidence of impact on performance was noted.

Thus, the overall effect of time was much more pronounced than any treatment effect on sow blood measurements, and all changes appeared to be mainly physiological and related to timing around farrowing, with no clinical manifestations of concern for nitrate toxicity.

### Reproductive Performance Variables

Reproductive performance variables were collected as secondary outcome variables (Table 6). Supplementation with nitrate tended to exert a quadratic effect on both percentage of piglets live born and stillborn (*P* < 0.10). Both nitrate supplemented treatment groups had higher percentages of live births. The percentage of live-born piglets was highest for the 1,200 ppm treatment group (97.36%), as compared to the control diet (92.52%) and 6,000 ppm (95.26%). The nitrate-supplemented groups had similar findings for proportion of stillborn piglets. Both treatment groups had lower percentages of stillborns, with the 1,200 ppm treatment having the lowest percentage of 2.55% and the 6,000 ppm treatment having 3.87% compared to the control diet with 7.17% stillborn.

**Table 6.** The effect of dietary nitrate supplementation on the percentage total born, liveborn, and stillbirth, and effect on birth and weaning weights

Variable	Treatment means			SEM	Contrast prob *F*	
	0 ppm	1,200 ppm	6,000 ppm		Linear	Quadratic
Live born, %	92.52	97.36	95.26	2.620	0.794	0.076
Stillborn, %	7.17	2.55	3.87	2.726	0.553	0.090
Mummies, %	1.27	1.43	0.763	1.003	0.624	0.814
Weaned pigs, % (after cross-fostering)	96.49	94.73	96.49	2.091	0.822	0.475
Birth weight, kg	1.23	1.26	1.24	0.057	0.932	0.647
Weaning weight, kg	6.16	5.64	5.87	0.168	0.543	0.033
Total born, *n*	19.6	17.5	16.4	—	—	—
Total live born, *n*	18.0	17.0	15.5	—	—	—
Total stillborn, *n*	1.6	0.5	0.8	—	—	—

The birth weight of piglets was similar across all treatments (*P* > 0.10), with weaning weights exhibiting a quadratic effect where the 1,200 ppm treatment had the lowest weaning weight (*P* < 0.05). The lower piglet weight for the 1,200 ppm treatment was first observed during the period from birth to cross-fostering, when the 1,200 ppm treatment had the lowest piglet average daily gain of 0.101 kg/d, whereas the zero and 6,000 ppm treatments had an average daily gain of 0.124 and 0.124 kg/d, respectively (*P* < 0.05; data not shown). Thus, the weight advantage for the zero and 6,000 ppm treatments was maintained from cross-fostering through weaning.

### Additional Performance Variables

Sow weights and backfat depth were similar across treatments at the beginning and end of the study (*P* > 0.10). As expected, sows lost approximately 8 to 10% of their body weight and 14 to 19% of their backfat due to the high energy demands of lactation (Cozannet et al., 2018; Houde et al., 2010). No treatment-related effect was present (Table 7).

**Table 7.** Effect of dietary nitrate supplementation on sow weight loss during lactation

Variable	Treatment means			SEM	Contrast prob *F*	
	0 ppm	1,200 ppm	6,000 ppm	SEM	Linear	Quadratic
Sow Entry Weight, ~D108 Gestation, kg	274.2	283.0	278.8	18.47	0.872	0.451
Sow Weaning Weight, kg	221.5	235.3	230.9	20.47	0.540	0.154
Sow Entry Backfat, ~D108 Gestation, mm	19.2	21.2	20.7	1.15	0.531	0.217
Sow Backfat at Weaning, mm	15.5	18.2	17.1	1.08	0.587	0.106

While this study collected data on performance variables in order to assess the safety profile of calcium nitrate (i.e., to ensure that meaningful deviations from expected performance did not occur), the focus of this study was not performance due to the limited number of replications. An opportunity area is to continue to understand the potential benefits of nitrate on performance variables indicated by van den Bosch (2019a, b) because the current work suggests no adverse effects at low levels of supplementation.

### Implications for Safety

Dietary nitrate has traditionally been considered a contaminant of concern in foods for humans as well as swine (Wikoff et al., 2018; Doepker et al., 2021). Reference documents such as the National Research Council Nutrient Requirements of Swine discuss low-level nitrate (290 to 490 ppm) in well water as being associated with toxicity (NRC, 2012). However, nitrate has begun to be recognized for favorable physiological effects and has been considered beneficial in the diets of both humans and swine (Hord et al., 2009; van den Bosch et al., 2019a, b). The reviews of Wikoff et al. (2018) in humans and Doepker et al. (2021) in swine suggest that the old case reports were confounded (potential bacterial contamination in well water) and lacking in detail. In fact, more recent work suggests that levels well above the 490 ppm value are safe in swine, up to ~600 to 800 mg/kg-bw/d (Doepker et al., 2021).

Calcium nitrate has been evaluated recently as a beneficial nitrate source in animal feed to improve reproductive outcomes in sows and their offspring when administered from day 108 of gestation until day 5 of lactation (van den Bosch et al., 2019a, b). Treatment diets in the work performed by van den Bosch et al., contained 0.00%, 0.03%, 0.06%, 0.09%, 0.12%, and 0.15% of dietary nitrate in the form of calcium nitrate and indicated maximum benefits between 0.09% and 0.12%. Current findings support the inclusion of nitrate levels 5 times greater than the target 1,200 ppm dose was associated with no clinical signs of nitrate poisoning in sows or piglets. While the level of methemoglobin tended to increase as the level of nitrate supplementation increased, the methemoglobin levels were well below 10%, with the highest measured hemoglobin level being 3.6%.

The current study confirms the hypothesis that supplementing up to 6,000 ppm nitrate in the diet from calcium nitrate is not associated with an increased risk of methemoglobinemia in lactating sows and nursing piglets. The analytical data for methemoglobin in the blood for both sows and piglets did not approach any threshold of toxic concern. Observational data and blood parameter values were generally within, or very near, expected ranges for healthy sows and piglets. Further, the secondary outcome percent of live born pigs provides further support that dietary nitric oxide precursors, such as calcium nitrate, may be conditionally essential during the periparturient period for sows with large litters.
